# SARS-CoV-2 Seroprevalence, Illness Recall, and Work Absenteeism Among Healthcare Workers: A Facility-Based Cross-Sectional Study in the Philippines, 2021

**DOI:** 10.1093/cid/ciaf342

**Published:** 2025-07-23

**Authors:** Maria Tricia Subido-Cariño, Morgane Donadel, Lea D Asi, Mary Shiela Ariola-Ramos, Vina Lea Arguelles, Stephanie N Co, Garrett Mahon, Sergie Paul C Fernandez, Kenneth Aristotle Punzalan, Johanna Beulah Sornillo, Rachel Smith, Matthew Westercamp, Ruth Anne Hechanova-Cruz

**Affiliations:** Medical Department, Research Institute for Tropical Medicine, Manila, Philippines; Department of Pediatrics, Cardinal Santos Medical Center, Manila, Philippines; Division of Healthcare Quality Promotion, US Centers for Disease Control and Prevention, Atlanta, Georgia, USA; Medical Department, Research Institute for Tropical Medicine, Manila, Philippines; Department of Medicine, Cardinal Santos Medical Center, Manila, Philippines; Medical Department, Research Institute for Tropical Medicine, Manila, Philippines; Department of Medicine, Cardinal Santos Medical Center, Manila, Philippines; Division of Healthcare Quality Promotion, US Centers for Disease Control and Prevention, Atlanta, Georgia, USA; Department of Medicine, Cardinal Santos Medical Center, Manila, Philippines; Pathology Department, Research Institute for Tropical Medicine, Manila, Philippines; Department of Biostatistics and Epidemiology, Research Institute for Tropical Medicine, Manila, Philippines; Division of Healthcare Quality Promotion, US Centers for Disease Control and Prevention, Atlanta, Georgia, USA; Division of Healthcare Quality Promotion, US Centers for Disease Control and Prevention, Atlanta, Georgia, USA; Medical Department, Research Institute for Tropical Medicine, Manila, Philippines

**Keywords:** COVID-19, surveillance, healthcare workers, patient safety, seroepidemiologic study

## Abstract

**Background:**

Healthcare workers (HCWs) have been disproportionately affected by coronavirus disease 2019 (COVID-19). This study assessed HCW severe acute respiratory syndrome coronavirus 2 (SARS-CoV-2) seroprevalence trends and indicators' effectiveness for infection monitoring in the Philippine healthcare setting during 2021 and 2022–2023.

**Methods:**

We obtained data on HCW demographics at 2 facilities, and COVID-19 symptoms recall and absenteeism at one facility. We collected and tested blood specimens with a SARS-CoV-2 spike and nucleocapsid protein–based serological assay. We calculated crude prevalence ratios (PRs) and 95% confidence intervals (CIs) for risk factors.

**Results:**

A total of 253 HCWs were included. Seroprevalence of SARS-CoV-2 infection among HCWs was 34% from September to October 2021 and 92% from October 2022 to April 2023 during the Delta and the Omicron variant surges, respectively, at one facility. At the other facility, seroprevalence was 31% in September–October 2021. Of the 33 seropositive HCWs with questionnaire completed, 14 (42%) did not recall COVID-19-like symptoms in the past year. Serological evidence of SARS-CoV-2 infection was statistically more likely among HCWs who recalled COVID-19-like symptoms in the past year (PR, 2.69 [95% CI: 1.53–4.72]; *P* < .05). Of the seropositive HCWs, 7 (21%) had no hospital documentation of missed work. Of the 24 HCWs with a positive SARS-CoV-2 test, 12 (50%) tested because of symptoms, 4 (17%) tested after exposure, and 5 (21%) tested following employment screening.

**Conclusions:**

Self-reported symptoms may be used to track SARS-CoV-2 prior infections within resource-limited healthcare settings. Occupational health services should consider these findings when developing infection monitoring strategies among HCWs. Combining surveillance strategies may strengthen infection monitoring.

## Teaser

This study aims to inform personnel in resource-limited healthcare settings about effective strategies for identifying SARS-CoV-2–positive healthcare workers. Illness recall may represent a useful monitoring approach to track SARS-CoV-2 infections within healthcare settings despite incomplete ascertainment.

Healthcare workers (HCWs) have experienced disproportionately high morbidity and mortality during the coronavirus disease 2019 (COVID-19) pandemic, particularly in low- and middle-income countries (LMICs) where healthcare infrastructure is already under strain [1]. The personal, physical, and psychological burdens placed on HCWs during this time have been widely reported [2–6]. In LMICs, these challenges are further exacerbated by resource shortages, making the loss of HCWs through isolation, quarantine, illness, or death particularly destabilizing to the healthcare system. The absence of HCWs, in turn, impacts patient care and related health outcomes, creating a critical need to balance staff safety with the provision of essential services [7]. This balance is complicated by the phenomenon of HCW presenteeism, where HCWs may continue working while symptomatic, suspecting an infection, or following high-risk exposures, increasing the risk of transmission [8].

In response to the pandemic, the Philippines, a middle-income country in Southeast Asia, launched a COVID-19 vaccination campaign starting on 1 March 2021. Despite this, vaccine uptake remained low with significant vaccine hesitancy reported nationwide [9–11]. By September 2021, during the severe acute respiratory syndrome coronavirus 2 (SARS-CoV-2) Delta variant surge, the Philippines had one of the highest rates of newly confirmed COVID-19 cases globally, while only 30% of the population had been fully vaccinated compared to 43% of the world population [9–12]. This combination of high transmission and low vaccination coverage presented a unique challenge in controlling the spread of SARS-CoV-2, particularly among HCWs, who were at increased risk of exposure.

Seroprevalence studies, which measure pathogen-specific antibodies in the serum, have been widely used during the COVID-19 pandemic to estimate seropositivity from infection and/or vaccination within populations. SARS-CoV-2 seroprevalence surveys provide valuable insight into population-level immunity and are useful tools for program planning and decision-making in public health [13–15]. While many early seroprevalence surveys were conducted in high-income countries [16], the seroprevalence of SARS-CoV-2 in LMICs remained unclear.

In addition, at the time of the study, there was limited understanding of the effectiveness of monitoring approaches that rely on self-recognition and reporting symptoms to identify COVID-19 infections among HCWs [17]. This study aimed to address these gaps by documenting seroprevalence trends among HCWs and assessing the utility and effectiveness of COVID-19 symptoms recall and work absenteeism as proxy measures for monitoring SARS-CoV-2 infections in healthcare settings.

## MATERIALS AND METHODS

### Study Sites and Population

The SARS-CoV-2 HCW Seroprevalence, Illness Recall, and Absenteeism Study (HCW-SIRAS) was designed as a rapid cross-sectional assessment of individual- and occupational-level monitoring approaches for identifying SARS-CoV-2–positive HCWs conducted at Cardinal Santos Medical Center (CSMC). CSMC is a private 300-bed tertiary care hospital located in Manila, the Philippines, offering a wide range of medical services, technology, and specialty centers. Participants at CSMC were HCWs engaged in direct patient care (eg, physicians, nurses, medical technicians) for at least 6 months prior to enrollment.

Due to limited HCW recruitment related to increasing pandemic response activities at CSMC, the Research Institute for Tropical Medicine (RITM) was considered a secondary study site to help increase the robustness and representativeness of seroprevalence estimates. RITM is a Philippine Department of Health facility, including the national tropical medicine and infectious disease research laboratory, multiple national reference laboratories, and a 50-bed specialty hospital for treating communicable diseases. Participants at RITM were HCWs engaged in direct patient care or patient specimen testing for at least the previous 6 months.

Due to questions regarding the interpretation of serologic results for individuals who received vaccines that may generate both anti-spike protein (S) and anti-nucleocapsid protein (N) responses, individuals reporting receipt of an inactivated COVID-19 vaccine (eg, Sinovac CoronaVac) were excluded from the study [18]. All other COVID-19 vaccines in use in the Philippines at the time (eg, Oxford-AstraZeneca, Moderna, Pfizer) were accepted into the study.

### Enrollment and Data/Specimen Collection

#### Cardinal Santos Medical Center

At CSMC, participant enrollment was conducted from September to October 2021. Eligible HCWs were identified through the hospital's human resources department and approached via email and in-person meetings to explain the study objectives and procedures. Informed consent was obtained from each participant before enrollment.

Participants from CSMC completed a questionnaire collecting basic demographics, occupation, vaccination history, and self-reported history of COVID-19 and SARS-CoV-2 testing in the prior 12 months. Following completion of the questionnaire, participants provided 5 mL of venous blood for serologic testing.

Occupational health data documenting HCW absenteeism were available as a COVID-19 census maintained by the CSMC Infection Control Committee. These data were linked to survey responses and serologic results for each participant enrolled.

#### Research Institute for Tropical Medicine

At RITM, participant enrollment occurred from October 2022 to April 2023, with a single group of HCWs enrolled throughout this period. HCWs were informed about the study through internal communication channels, including email and bulletin boards. Informed consent was obtained from each participant before enrollment.

As part of an existing RITM program monitoring staff SARS-CoV-2 serology, banked blood specimens were available for some staff enrolled. When banked blood specimens collected between September and October 2021 were available, they were utilized for this study. For all other participants, a 5 mL venous blood specimen was collected. Due to the use of banked blood samples at RITM and the difference in the recall period for questionnaire responses between CSMC and RITM participants, we only considered and included demographic characteristics that were unlikely to vary over the period from questionnaire to banked blood sample collection (eg, occupation, assigned patient care area) in the data analyses of RITM participants. Each study participant was tested with a SARS-CoV-2 serology assay once, either during September–October 2021 or during October 2022–April 2023. Serologic results were linked to survey responses.

### Blood Specimen Testing and Seroprevalence Outcome Measures

Serum specimens were tested for anti-S and anti-N antibody levels by electrochemiluminescence Elecsys Anti-SARS-CoV-2 S and Elecsys Anti-SARS-CoV-2 N immunoassays (Roche Diagnostics, Mannheim, Germany) using the Roche Cobas e411 platform (Roche Holding, Basel, Switzerland). Infection-induced seroprevalence was defined as the prevalence of the population positive for both SARS-CoV-2 S and N antibodies. Using this definition, seropositive participants were defined as positive for N and S while seronegative participants were defined as negative for N and either positive or negative for S. In contrast, seroprevalence due to either infection or vaccination was defined as the prevalence of the population positive for S antibodies. Using that definition, seropositive participants were defined as positive for S while seronegative participants were defined as negative for S. Seropositive and seronegative participants due to either infection or vaccination could be either positive or negative for N.

Banked serum specimens were stored at −20°C and study-specific specimens were banked until testing. Specimens were thawed and centrifuged prior to testing, following assay instructions.

### Data Management and Analysis

All study data were entered and managed in a REDCap database [19].

Prevalence ratios (PRs) for infection-induced seroprevalence were calculated using a log binomial model to determine the significant differences in categorical participant demographics at both study sites and in vaccination history, illness recall, and work absenteeism characteristics at CSMC. Results were expressed as crude PRs and 95% confidence intervals (CIs). All tests performed were 2-sided, a *P* value of <.05 was considered statistically significant, and data analyses were conducted using R software (version 4.1.3) [20].

### Ethical Considerations

The protocol and all study activities were reviewed and approved by the CSMC and RITM institutional review boards. This activity was also reviewed by the US Centers for Disease Control (CDC) and conducted according to applicable federal law and CDC policy as defined in 45 Code of Federal Regulations 46.102(I) [21].

## RESULTS

### Enrollment and Participant Characteristics

At CSMC, 109 HCWs participated in the study, with 106 (97%) providing a whole blood specimen for SARS-CoV-2 serologic testing.

The median age of participants at CSMC was 35 years, with a majority being female HCWs (64% [67/106]) (Table 1). Most enrolled HCWs were primarily assigned to patient care wards not specifically designated for the care of COVID-19 patients (76% [81/106]) and were predominantly physicians (66% [70/106]), followed by nurses (11% [12/106]), non clinicians (7% [7/106]), and other clinical professions (eg, physical therapist) (1% [3/106]). Twenty-five percent of the enrolled HCWs (27/106) at CSMC also worked at another facility. Most (94% [100/106]) perceived their health as excellent or good.

**Table 1.** Demographics of Cardinal Santos Medical Center Participants Enrolled by Severe Acute Respiratory Syndrome Coronavirus 2 Infection–Induced Seropositivity Status, September–October 2021

Characteristic^a^	Total	Seropositive^b^	Seronegative^c^	PR (95% CI)	*P* Value
Total	106 (100)	33 (31)	73 (69)		
Age, y, median (IQR)		33 (29–40)	37 (30–51)		.13
Age					
<35 y	49 (100)	20 (41)	29 (59)	1.79 (1.00–3.21)	.17
≥35 y	57 (100)	13 (23)	44 (77)		
Sex					
Female	67 (100)	21 (31)	46 (69)	1.08 (.59–2.00)	.86
Male	38 (100)	11 (29)	27 (71)		
Primary assigned patient care area					
COVID-19 wards	25 (100)	9 (36)	16 (64)	1.22 (.65–2.26)	.69
Patient care wards not designated for the care of COVID-19 patients	81 (100)	24 (30)	57 (70)		
Occupation					
Other clinical profession	3 (100)	0 (0)	3 (100)	0.00	>.99
Non clinician	7 (100)	2 (29)	5 (71)		
Nurse	12 (100)	5 (42)	8 (67)	1.35 (.35–5.24)	.77
Physician	70 (100)	23 (33)	47 (67)	1.15 (.34–3.89)	.87
Working in multiple healthcare facilities					
Yes	27 (100)	8 (30)	19 (70)	0.90 (.46–1.75)	.83
No	76 (100)	25 (33)	51 (67)		

Data are presented as No. (%) unless otherwise indicated.

Abbreviations: CI, confidence interval; COVID-19, coronavirus disease 2019; IQR, interquartile range; PR, prevalence ratio.

^a^Data reported exclude “Unknown” categories. The log binomial model tests that the distribution of the study participants was different between the seropositive and seronegative groups, excluding “Unknown” categories.

^b^Seropositive is defined as positive for nucleocapsid protein (N) and spike protein (S) antibodies.

^c^Seronegative is defined as negative for N and either positive or negative for S. Of the 73 seronegative healthcare workers, 1 was S negative while all the others were S positive.

**Figure 1.**
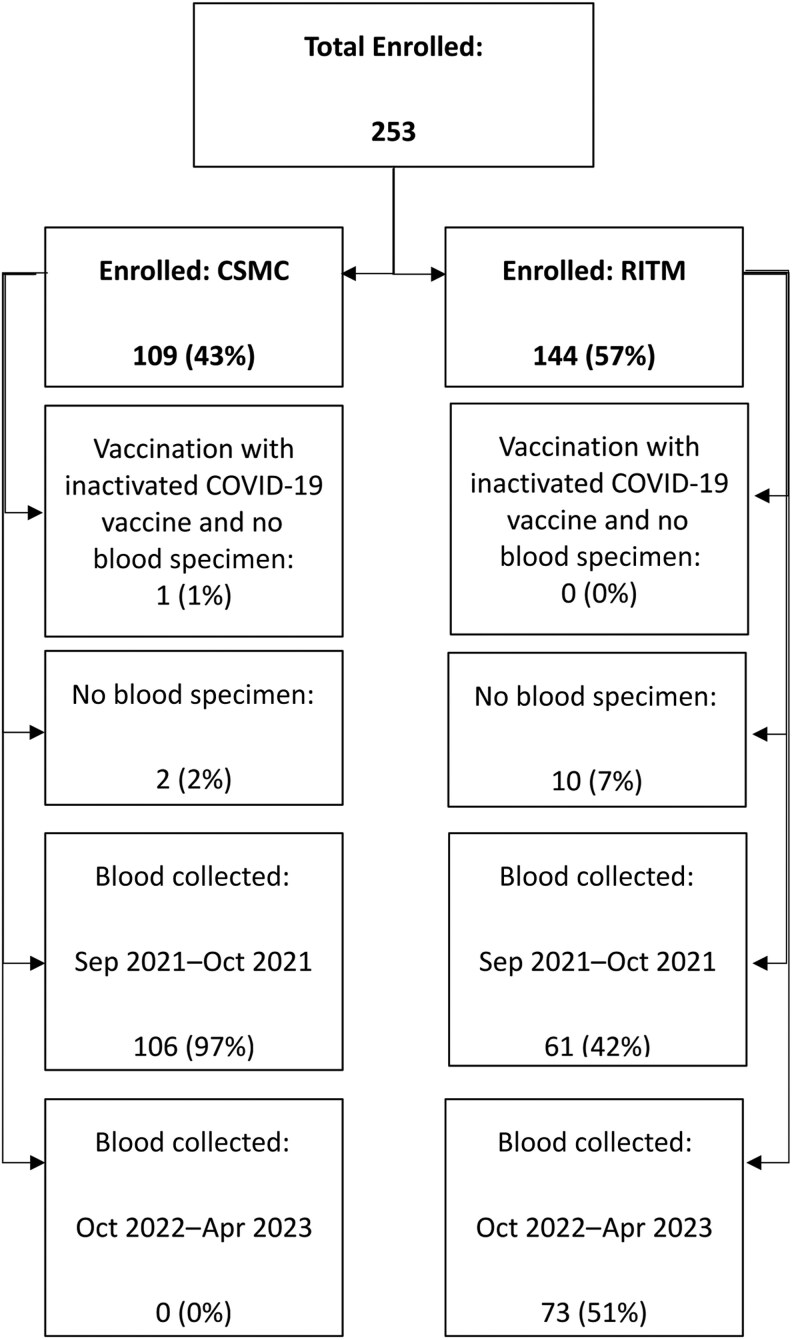
Participant enrollment and blood specimen collection by study site, SARS-CoV-2 Seroprevalence, Illness Recall, and Work Absenteeism Among Healthcare Workers (HCW-SIRAS) study, 2021–2023. Abbreviations: COVID-19, coronavirus disease 2019; CSMC, Cardinal Santos Medical Center; RITM, Research Institute for Tropical Medicine.

At RITM, 144 HCWs agreed to participate in the study. Of these, 61 (42%) had banked serum specimens collected during the period comparable to the CSMC sample collection (September–October 2021) (Figure 1). Seventy-three (51%) participants had their blood specimens collected between October 2022 and April 2023. Ten (7%) had no blood specimen collected.

The median age of participants at RITM was 34.5 years, with a majority being female HCWs (69% [93/134]) (Table 2). Most participants were primarily assigned to patient care wards not specifically designated for the care of COVID-19 patients (87% [114/134]) and were predominantly non clinicians (37% [49/134]), a group made up largely of administrative staff, laboratorians, and researchers. Nurses accounted for 17% (23/134), other clinical professions for 10% (14/134), and physicians for 3% (4/134). Only a small proportion (4% [5/134]) of the enrolled HCWs at RITM also worked at another facility. More than 95% (128/134) of them perceived their health as excellent or good.

**Table 2.** Demographics of Research Institute for Tropical Medicine Participants Enrolled by Severe Acute Respiratory Syndrome Coronavirus 2 Infection–Induced Seropositivity Status, September–October 2021 and October 2022–April 2023

Characteristic^a^	RITM 2021					RITM 2022–2023				
	Total	Seropositive^b^	Seronegative^c^	PR (95% CI)	*P* Value	Total	Seropositive^b^	Seronegative^d^	PR (95% CI)	*P* Value
Total	61 (100)	21 (34)	40 (66)			73 (100)	67 (92)	6 (8)		
Age										
<35 y	37 (100)	11 (30)	26 (70)	0.71 (.36–1.42)	.54	30 (100)	28 (93)	2 (7)	1.03 (.90–1.18)	.97
≥35 y	24 (100)	10 (42)	14 (58)			43 (100)	39 (91)	4 (9)		
Sex										
Female	42 (100)	17 (40)	25 (60)	1.92 (.75–4.95)	.31	51 (100)	46 (90)	5 (10)	0.94 (.83–1.07)	.96
Male	19 (100)	4 (21)	15 (79)			22 (100)	21 (95)	1 (5)		
Primary assigned patient care area										
COVID-19 wards	1 (100)	1 (100)	0 (0)	3.00 (2.10–4.29)	>.99	16 (100)	15 (94)	1 (6)	1.44 (.21–28.8)	.75
Patient care wards not designated for the care of COVID-19 patients	60 (100)	20 (33)	40 (67)			57 (100)	52 (91)	5 (9)		
Occupation										
Other clinical profession	1 (100)	1 (100)	0 (0)	3.88 (2.13–7.04)	>.99	13 (100)	13 (100)	0 (0)	1.20 (.98–1.48)	>.99
Non clinician	31 (100)	8 (26)	23 (74)			18 (100)	15 (83)	3 (17)		
Nurse	1 (100)	1 (100)	0 (0)	3.88 (2.13–7.04)	>.99	22 (100)	21 (95)	1 (5)	1.15 (.91–1.44)	.91
Physician	1 (100)	0 (0)	1 (100)	0.00	>.99	3 (100)	1 (33)	2 (67)	0.40 (.08–2.01)	.51
Working in multiple facilities										
Yes	0 (0)	0 (0)	0 (0)			5 (100)	4 (80)	1 (20)	0.86 (.55–1.35)	.90
No	61 (100)	21 (34)	40 (66)			68 (100)	63 (93)	5 (7)		

Data are presented as No. (%) unless otherwise indicated.

Abbreviations: CI, confidence interval; COVID-19, coronavirus disease 2019; PR, prevalence ratio; RITM, Research Institute for Tropical Medicine.

^a^Data reported exclude “Unknown” categories. The log binomial model tests that the distribution of the study participants was different between the seropositive and seronegative groups, excluding “Unknown” categories.

^b^Seropositive is defined as positive for nucleocapsid protein (N) and spike protein (S) antibodies.

^c^Seronegative is defined as negative for N and either positive or negative for S. Of the 40 seronegative healthcare workers with banked blood specimens, 1 was S negative while all the others were S positive.

^d^Seronegative is defined as negative for N and either positive or negative for S. All 6 seronegative healthcare workers with specimens collected during October 2022–April 2023 were S positive.

### SARS-CoV-2 Seroprevalence

Based on our sample, HCW infection-induced seroprevalence at CSMC in September–October 2021 was 31.1% (33/106) (95% CI: 22.7%–41.0%). At RITM, infection-induced seroprevalence during the same period was 34.4% (21/61) (95% CI: 23.0%–47.8%). By the period between October 2022 and April 2023, HCW infection-induced seroprevalence at RITM was 91.8% (67/73) (95% CI: 82.4%–96.6%), indicating a substantial rise in seroprevalence from 2021 (*P* < .001).

HCW seroprevalence due to infection or vaccination at CSMC in September–October 2021 was 99.1% (105/106) (95% CI: 94.1%–100%). At RITM, seroprevalence due to infection or vaccination during the same period was 98.4% (60/61) (95% CI: 90.0%–99.9%). By the period between October 2022 and April 2023, HCW seroprevalence due to infection or vaccination at RITM was 100% (73/73) (95% CI: 93.8%–100%).

### Factors Associated With HCW SARS-CoV-2 Infection–Induced Seroprevalence

#### Demographics of CSMC and RITM Participants

There was no significant statistical association between age, sex, primary assigned hospital ward, or the HCW occupation with serological evidence of prior infection among CSMC participants during 2021 (Table 1) or among RITM participants during 2021 or 2022–2023 (Table 2).

#### Vaccination History, Patient Care, and Workplace Safety of CSMC Participants

Almost all (97% [32/33]) HCWs at CSMC with serological evidence of prior infection had received 2 doses of the COVID-19 vaccine at time of enrollment, with the majority (91% [29/32]) having received the Oxford-AstraZeneca vaccine. Most participants (79% [26/33]) had provided care to a suspected or confirmed COVID-19 patient as part of their job, and the majority (85% [28/33]) felt safe from contracting SARS-CoV-2 infection at work. No significant statistical association was observed between COVID-19 vaccination, providing care to a COVID-19 patient, or perceived workplace safety and serological evidence of prior infection among participants from CSMC (Table 3).

**Table 3.** Vaccination History, Patient Care Provided, Perceived Workplace Safety, Illness Recall, Severe Acute Respiratory Syndrome Coronavirus 2 (SARS-CoV-2) Testing Conducted, and Missed Work Documentation Among Cardinal Santos Medical Center Participants Enrolled by SARS-CoV-2 Infection–Induced Seropositivity Status, September–October 2021

Characteristic^a^	Total	Seropositive^b^	Seronegative^c^	PR (95% CI)	*P* Value
Total	106 (100)	33 (31)	73 (69)		
Received ≥1 dose of COVID-19 vaccine					
Yes	104 (100)	32 (31)	72 (69)	0.62 (.15–2.53)	.73
No	2 (100)	1 (50)	1 (50)		
Received 2 doses of COVID-19 vaccine					
Yes	103 (100)	32 (31)	71 (69)	0.62 (.15–2.56)	.74
No	2 (100)	1 (50)	1 (50)		
Provided care to a suspected or confirmed COVID-19 patient in the past year					
Yes	77 (100)	26 (34)	51 (66)	1.55 (.67–3.58)	.43
No	23 (100)	5 (22)	18 (78)		
Felt safe and able to follow facility safety/PPE protocol for each activity, when providing direct patient care to COVID-19 suspected or confirmed patients over the past year					
Always/most of the time	81 (100)	28 (35)	53 (65)		
Occasionally	1 (100)	0 (0)	1 (100)	0.00	>.99
Felt safe from contracting COVID-19 infection at work over the past year					
Yes/mostly	82 (100)	26 (32)	56 (68)	3.80 (.57–25.52)	.21
No	12 (100)	1 (8)	11 (92)		
Recalled illness that prevented or should have prevented from going to work in the past year					
Yes	33 (100)	18 (55)	15 (45)	2.69 (1.53–4.72)	.03
No	69 (100)	14 (20)	55 (80)		
Tested positive for SARS-CoV-2 or were told to have COVID-19 in the past year					
Yes	25 (100)	19 (76)	6 (24)	4.29 (2.54–7.24)	.009
No	79 (100)	14 (18)	65 (82)		
Documented as having missed work within the last year in occupational health records					
Yes	67 (100)	26 (39)	41 (61)	2.16 (1.04–4.51)	.11
No	39 (100)	7 (18)	32 (82)		

Data are presented as No. (%) unless otherwise indicated.

Abbreviations: CI, confidence interval; COVID-19, coronavirus disease 2019; CSMC, Cardinal Santos Medical Center; PPE, personal protective equipment; PR, prevalence ratio; SARS-CoV-2, severe acute respiratory syndrome coronavirus 2.

^a^Data reported exclude “Unknown” category. The log binomial model tests that the distribution of the study participants was different between the seropositive and seronegative groups, excluding “Unknown” categories.

^b^Seropositive is defined as positive for nucleocapsid protein (N) and spike protein (S) antibodies.

^c^Seronegative is defined as negative for N and either positive or negative for S. Of the 73 seronegative healthcare workers, 1 was S negative while all the others were S positive.

#### Previous Illnesses and SARS-CoV-2 Testing of CSMC Participants

Of the 33 CSMC HCWs with serological evidence of prior infection, 14 (42%) did not recall any illness in the past year that kept them from going to work or should have kept them from going to work. Serological evidence of SARS-CoV-2 infection was statistically more likely among CSMC HCWs who recalled an illness in the past year (PR, 2.69 [95% CI: 1.53–4.72]; *P* < .05) and among those who reported testing positive for SARS-CoV-2 (PR, 4.29 [95% CI: 2.54–7.24]; *P* < .05) (Table 3).

Of the 24 CSMC HCWs with a respiratory specimen positive for SARS-CoV-2 and data available, 12 (50%) were tested because of symptoms while 4 (17%) tested because of an exposure to a COVID-19 case, 2 (8%) tested because of symptoms and exposure, 5 (21%) tested following employment screening, and 1 (4%) just wanted to be tested.

#### Absenteeism From Work of CSMC Participants

Of the 33 HCWs with serological evidence of prior infection, 26 (79%) had documentation of missed work. In comparison, 41 of the 73 (56%) HCWs without serological evidence of prior infection had missed work. No significant statistical association was observed between documentation of missed work in the facility occupational health records and serological evidence of prior infection among CSMC participants (Table 3).

## DISCUSSION

In this cross-sectional study of HCWs, we described recall of COVID-19 symptoms, SARS-CoV-2 testing, and work absenteeism using occupational health records. We also assessed individual-level characteristics associated with serological evidence of SARS-CoV-2 prior infection among HCWs at 2 hospitals in the Philippines. The infection-induced seroprevalence among HCWs was similar at both hospitals in September–October 2021 and showed an increase by October 2022–April 2023 following the Omicron surge at one of the hospitals. This observed change in infection-induced seroprevalence was consistent with those documented in the general population during the same period in other countries in the Western Pacific region (eg, China, Japan) [16]. Neither HCW demographics nor occupation were associated with serological evidence of infection in either time period, which suggests that the burden of transmission occurred at the community level at the time.

We also explored the association between HCWs' recall of illness, SARS-CoV-2 testing, and work absenteeism, using occupational health records alongside serological evidence of SARS-CoV-2 infection in 2021 at a Philippine hospital. This study, developed in response to specific resource constraints during the COVID-19 pandemic, aimed to inform occupational health services and infection prevention and control teams in resource-limited healthcare settings about effective strategies for identifying SARS-CoV-2–positive HCWs.

During September–October 2021, HCWs at CSMC who recalled an illness in the past year or tested positive for SARS-CoV-2 were statistically more likely to show serological evidence of prior infection. However, the sensitivity of illness recall for detecting prior infection was moderate, with 56% of seropositive HCWs recalling symptoms. Additionally, the positive predictive value of symptom recall was similar, as only 55% of those recalling symptoms were seropositive. This suggests that while self-reported symptoms may provide some indication of prior infection, they are not a highly reliable proxy. In this context, public health messages like “don’t come to work if you are sick” remain relevant strategies for reducing COVID-19 transmission, but they may not be sufficient as standalone detection tools in healthcare settings.

Conversely, negative predictive value was higher, with 80% of those who did not recall symptoms being seronegative. This suggests that the absence of symptom recall was more indicative of true seronegativity, although 20% of individuals not recalling symptoms were seropositive. The substantial proportion of unrecognized infections (42% of seropositive HCWs) could be attributed to mild or asymptomatic cases, in a predominantly vaccinated population, with preexisting immunity, and with relatively low prevalence of comorbidities that increase the risk of severe COVID-19.

Symptom recognition remained the most common reason for SARS-CoV-2 testing, far exceeding occupational screening. Given the limitations of symptom recall for identifying infections, occupational screening strategies, such as routine or targeted testing, may be beneficial for identifying asymptomatic cases and mitigating the impact of presenteeism.

Additionally, 21% of HCWs with serological evidence of prior infection had no documented work absenteeism in occupational health records, suggesting the complex interplay between symptom self-assessment and work behavior. This could reflect unrecognized infections, lack of symptom manifestation, or presenteeism among infected individuals. Although no significant statistical association was observed between seroprevalence and absenteeism, these findings highlight the need for further evaluation of absenteeism tracking as a supplemental surveillance tool, particularly during high transmission periods, to better understand infection transmission patterns among HCWs.

While illness recall may provide some insight into prior SARS-CoV-2 infections, it is not a comprehensive surveillance measure on its own. A combined approach, integrating self-reported symptoms with occupational health screening, could improve infection monitoring in healthcare settings. Educational efforts to lower the threshold for self-identifying as ill and increasing access to testing may also help improve detection.

There are several limitations to this study. First, the findings are specific to the facilities and participants involved and may not be generalizable to other settings. The potential for selection bias by excluding participants reporting receipt of an inactivated COVID-19 vaccine may have affected the accuracy of our findings. The lack of statistical power may explain the lack of association found between risk factors and SARS-CoV-2 seroprevalence. Second, while we supplemented self-reported illness with occupational health records, these records may have been incomplete, particularly if work absences were not accurately documented. The potential for recall bias could have affected the accuracy of self-reported illness. Third, seroprevalence studies also have limitations. Antibody levels may wane over time, leading to underestimation of prior infections, especially in highly vaccinated individuals. Additionally, serological testing cannot differentiate between mild and severe infections, making it less useful for understanding the full clinical picture. Fourth, incomplete absenteeism data could have skewed the use of occupational health records as reliable infection indicators, suggesting the true infection burden may have been higher than observed. Fifth, each study participant was tested with a SARS-CoV-2 serology assay once, either during September–October 2021 or during October 2022–April 2023. While the seroprevalence estimates and observed change between the two time periods are consistent with those found in the literature [16], using a one-time-point serological assay limited our ability to fully appreciate trends in SARS-CoV-2 seroprevalence over time. Finally, this study design did not allow us to fully assess the utility and return of investment of occupational screening to prevent transmission in healthcare settings.

## CONCLUSIONS

This study contributes to the limited data on SARS-CoV-2 seroprevalence in the Philippines. Similar to findings in other Western Pacific countries, a substantial increase in infection-induced seroprevalence was observed following the emergence of the Omicron variant. While illness recall was associated with a higher probability of being seropositive, it demonstrated moderate sensitivity and low positive predictive value, indicating that it is not a reliable standalone measure for tracking prior SARS-CoV-2 infections. These findings suggest the need for combining surveillance strategies to enhance infection detection in healthcare settings. Insights from this study may help occupational health services and infection prevention teams refine their strategies for monitoring and mitigating COVID-19 transmission among HCWs.

## Notes

***Author contributions.*** M. T. S.-C. and R. A. H.-C. contributed to methodology, investigation, project administration, and editing of drafts. L. D. A., V. L. A., M. S. A.-R., S. N. C., S. P. C. F., and K. A. P. contributed to methodology, investigation, and editing of drafts. J. B. S. contributed to methodology, investigation, data curation, and editing of drafts. G. M. contributed to formal analysis and editing of drafts. R. S. and M. W. contributed to conceptualization, funding acquisition, methodology, project administration, supervision, and editing of drafts. M. D. contributed to formal analysis, writing of the original draft, and editing of drafts. All authors have made substantial contributions to the development of the manuscript, read and approved the final manuscript, and made the decision to submit the manuscript for publication.

***Acknowledgments.*** The authors would like to thank the healthcare workers enrolled in the study as well as all the laboratory technicians and researchers who have contributed to this study. We acknowledge Mardee Allen Balde, Jessica McLean, India Bowman, and Alle Taylor at Health Security Partners for supporting the project administration, Amado Tandoc at the Research Institute for Tropical Medicine for supporting the conceptualization and draft of the protocol, and Fernanda Lessa at the US Centers for Disease Control and Prevention (CDC) for supporting the study and reviewing the manuscript.

***Financial support.*** This project described was supported by the US Centers for Disease Control and Prevention (cooperative agreement 6 NU2HGH000036).
